# Causal relationships between the gut microbiota, inflammatory cytokines, and alcoholic liver disease: a Mendelian randomization analysis

**DOI:** 10.3389/fendo.2024.1442603

**Published:** 2024-10-21

**Authors:** Shanzheng Li, Cheng Zhou, Tong Liu, Lihui Zhang, Sutong Liu, Qing Zhao, Jiangkai Liu, Wenxia Zhao

**Affiliations:** ^1^ Department of Gastroenterology, First Affiliated Hospital of Henan University of Chinese Medicine, Zhengzhou, China; ^2^ The First Clinical Medical College of Henan University of Chinese Medicine, Zhengzhou, China; ^3^ Department of Gastroenterology, Changzhou Hospital of Traditional Chinese Medicine, Changzhou, China

**Keywords:** Mendelian randomization, gut microbiota, inflammatory cytokines, alcoholic liver disease, causal relationship

## Abstract

**Objective:**

Previous studies have suggested a potential association between gut microbiota and the development of alcohol-related liver disease (ALD). However, the causal relationship between gut microbiota and ALD, as well as the role of inflammatory cytokines as mediators, remains unclear. This study aims to explore the causal relationship between gut microbiota and ALD using Mendelian randomization (MR) methods, and to analyze the mediating role of inflammatory cytokines.

**Methods:**

Gut microbiota, 91 inflammatory cytokines, and ALD were identified from summary data of large-scale genome-wide association studies (GWAS). MR was employed to investigate the causal relationship between gut microbiota, cytokines, and ALD, with the inverse variance-weighted method (IVW) as the primary statistical approach. Additionally, we examined whether inflammatory cytokines act as mediating factors in the pathway from gut microbiota to ALD.

**Results:**

The IVW results confirmed two positive and one negative causal effect between genetic liability in the gut microbiota and ALD. *Escherichia coli* (*P*= 0.003) was identified as a protective factor for ALD, while *Roseburia hominis* (*P*=0.023) and *Family Porphyromonadaceae* (*P*=0.038) were identified as risk factors for ALD. Furthermore, there were five positive and two negative causal effects between inflammatory cytokines and ALD, with CUB domain-containing protein 1 (P= 0.035), Macrophage colony-stimulating factor 1 (P=0.047), Cystatin D (P = 0.035), Fractalkine (P=0.000000038), Monocyte chemoattractant protein-1 (P=0.004) positively associated with ALD onset. CD40L receptor (P= 0.044) and Leukemia inhibitory factor (P = 0.024) exhibited protective effects against ALD. Mediation MR analysis indicated that CUB domain-containing protein 1 (mediation proportion=1.6%, P=0.035), Cystatin D (mediation proportion=1.5%, P=0.012), and Monocyte chemoattractant protein-1 (mediation proportion=3.3%, P=0.005) mediated the causal effect of *Roseburia hominis* on ALD.

**Conclusion:**

In conclusion, our study supports a causal relationship among gut microbiota, inflammatory cytokines and ALD, with inflammatory cytokines potentially acting as mediating factors in the pathway from gut microbiota to ALD.

## 1 Introduction

Alcohol-related liver disease (ALD) refers to liver diseases primarily caused by long-term heavy alcohol consumption, which includes asymptomatic hepatic steatosis, fibrosis, cirrhosis, alcoholic hepatitis, and their complications. Approximately 8-20% of chronic alcohol drinkers develop ALD with cirrhosis, and about 2% progress to hepatocellular carcinoma (1). Epidemiological research has demonstrated that ALD accounts for approximately 19% of global mortality attributable to alcohol-related liver cancer, and up to 25% of deaths from cirrhosis secondary to alcohol related liver disease (2, 3). Most patients with ALD do not exhibit overt symptoms during the early inflammatory phase prior to the development of cirrhosis. However, once the disease progresses to the cirrhotic stage, they may develop serious clinical manifestations including ascites, gastrointestinal bleeding, and edema, which are associated with a poor overall prognosis. The onset of ALD is typically insidious, characterized by nonspecific clinical symptoms in the early stages. Existing evidence suggests the pathogenesis of alcoholic liver disease involves complex interactions between various cell types and organ systems. Importantly, modulation of the gut microbiota has emerged as a potential therapeutic target for ALD (4).

The liver possesses remarkable self-repair capabilities that rely on the coordinated function of diverse cell types and extracellular factors (5). Additionally, there is a well-established connection between the liver and intestines via the portal vein, the biliary system and circulating mediators, allowing for bidirectional microbial interactions between the gut and liver (6). Consequently, the crosstalk between the gut microbiome and the liver has received increasing attention in context (7).

The human gut microbiome is characterized by vast diversity, with the majority of bacteria belonging to the phyla Firmicutes (60-80%), Bacteroidetes (20-40%), Proteobacteria, Actinobacteria, Verrucomicrobia, Fusobacteria, and Cyanobacteria (8). Alterations in the relative abundance of these bacterial phyla have been shown to impact various dimensions of human health (9). Studies indicated that Patients with alcohol-use disorder and liver disease exhibit reduced gut bacterial diversity, with decreased proportions of several beneficial bacteria, including *Lactobacillus*, *Bifidobacterium*, *Prevotella*, and *Akkermansia muciniphila*. In addition, these patients demonstrate shifts in gut microbiome composition, with decreased fungal diversity and increased proportions and quantities of Candida (10). Specifically, Grander et al. found a decreased abundance of the symbiont A. mucinip hila in patients with ALD, and supplementation of this bacterium significantly improved ethanol-induced intestinal and liver damage in mice (11). Furthermore, Duan et al. discovered a correlation between the severity of ALD and the cellulase activity of fecal Enterococcus, and targeting these bacteria with bacteriophages reduced cellulase levels in the liver and ameliorated liver disease in humanized mouse models (12). In ALD, the gut microbiota promotes inflammation along the gut-liver axis, while the presence of inflammatory cytokines may further accelerate disease progression (13). Establishing a causal relationship between ALD and gut microbiome alterations has proven challenging in previous studies, owing to the influence of confounding factors such as ethanol exposure, inflammation, and the complex interplay between these variables.

To overcome the limitations of confounding and reverse causality inherent in observational epidemiological studies, elucidate the causal relationships between multiple factors in the progression of the disease condition. The Mendelian randomization (MR) method, using single nucleotide polymorphisms (SNPs) strongly associated with exposure as instrumental variables (IV), has been increasingly accepted and utilized to assess causal effects between exposure and outcomes (14). The genetic variations used as instrumental variables in MR are fixed at conception, allowing researchers to make causal inferences about the impact of modifiable risk factors while overcoming the influence of certain confounding factors (15). Early genetic studies have suggested that host genetic variations can influence the composition of the gut microbiota, providing a foundation for employing the MR method to investigate the causal relationship between gut microbiota and ALD, as well as the potential mediating role of inflammatory processes. In this study, we aim to leverage summary data from genome-wide association studies (GWAS) and implement a bidirectional and mediation MR design across two independent samples. This approach will enable us to explore the causal relationship between gut microbiota and ALD, as well as elucidate the mediating role of inflammatory factors in this relationship.

## 2 Materials and methods

### 2.1 Study design

**Figure 1:**
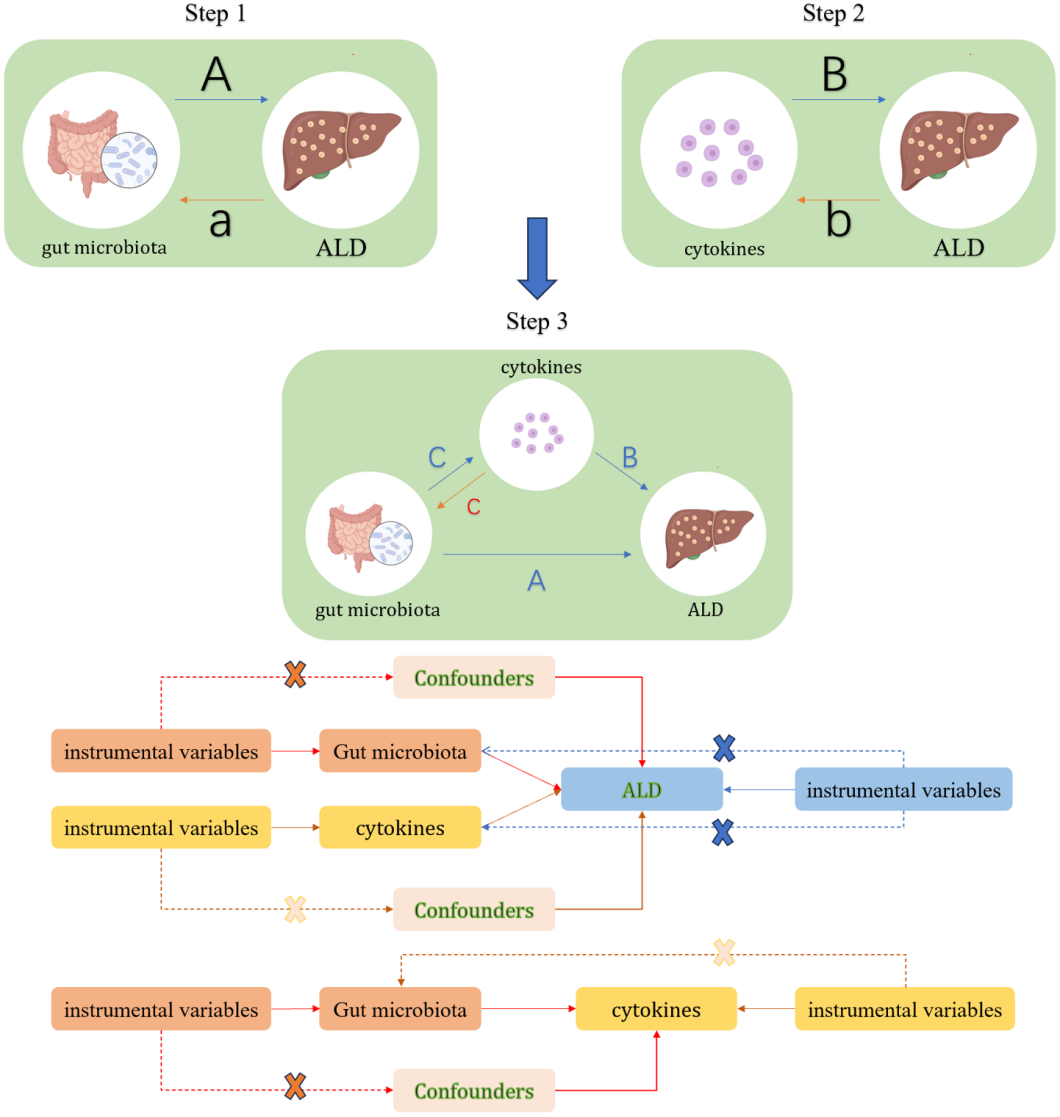
Study Overview. In Step 1, path A represents the causal impact of gut microbiota on ALD, while path a denotes the reverse causal effect of ALD on gut microbiota. In Step 2, path B signifies the causal impact of cytokines on ALD, with path b indicating the reverse causal effect between ALD and cytokines. Step 3 involves the mediation analysis of cytokines in the pathway from gut microbiota to ALD: Path A represents the overall impact of gut microbiota on ALD; Path B signifies the causal effect of cytokines on ALD; Path C indicates the causal effect of gut microbiota on cytokines, and path c denotes the reverse causal effect of cytokines on gut microbiota.

This study will consist of three main components. First, analyzing the causal effects of gut microbiota composition, comprising 207 distinct taxa, on the development of ALD using MR (Path A); second, investigating the causal impact of 91 inflammatory cytokines on the risk of ALD, also employing the MR approach (Path B); and third, examining the potential mediating role of the 91 cytokines in the causal pathway from gut microbiota composition to ALD (Path C). MR analysis will leverage SNPs as instrumental variables (IVs). This method relies on three core assumptions (16): (1) The selected SNPs must be strongly associated with the exposure factors of interest; (2) The SNPs must be independent of confounding factors; (3) The SNPs must influence the outcome only through their effect on the exposure, and not directly (Refer to **Figure 1** for a detailed flowchart of the study design) (16).

### 2.2 Data source

Genetic data on gut microbiome were obtained from the Dutch Microbiome Project (DMP) genome-wide association study (GWAS) of 7,738 individuals of Dutch ancestry (17, 18). The data included 207 taxonomic groups spanning 5 phyla, 10 classes, 13 orders, 26 families, 48 genera, and 105 species. Data on 91 inflammatory cytokines were sourced from a GWAS of 14,824 individuals of European descent across 11 cohorts, and these individuals are all from England (accession numbers GCST90274758 to GCST90274848) (19).

Large-scale GWAS meta-analysis data on ALD were obtained from the FinnGen consortium (https://www.finngen.fi/fi) (17). The FinnGen consortium is one of the largest GWAS databases globally, with a sample size of 2,408 available disease phenotypes, and it aims to study the genome and national health register data of 500,000 Finnish individuals. For this study, the ALD phenotype data were sourced from the FinnGen GWAS summary statistics, which are publicly available through the IEU OPEN GWAS PROJECT (https://gwas.mrcieu.ac.uk/), with the dataset coded as finn-b-K11_ALCOLIV.

This study involves secondary analysis of publicly available GWAS summary-level data, all of which have received the appropriate ethical approvals from the respective cohorts and consortia. As the study does not utilize individual-level participant data, no additional ethical review board approval is required.

### 2.3 Instrumental variables selection

Previous studies have demonstrated that independent SNPs form the basis of MR analyses (20). We selected SNPs with significant associations (P < 1×10^-5^) and an effect allele frequency (EAF) > 0.01 with gut microbiota and inflammatory protein factors to serve as instrumental variables. During the analysis, due to the limited number of inflammatory cytokines reaching the conventional genome-wide significance threshold of P < 5×10^-8^ for independent SNPs, the significance threshold was adjusted to P < 1×10^-5^. This was followed by linkage disequilibrium analysis (LDA) to ensure the independence of the selected SNPs, using a standard threshold of r^2^ < 0.1 within a 500kb window. An important step in MR analysis is ensuring that the effect allele for the association between the SNPs and the exposure corresponds to the effect allele for the association between the SNPs and the outcome. After this matching process, we removed any palindromic SNPs. (A palindromic SNP is an SNP with the A/T or G/C allele.) from the analysis.

### 2.4 MR analysis

#### 2.4.1 Primary analysis

To assess the causal impact of gut microbiota and inflammatory cytokines on ALD, we conducted analyses using the Two Sample MR (21), MRPRESSO, and MR packages in R studio (version 4.3.2). Initially, we performed MR analyses on two separate samples (steps A and B in **Figure 1**). During the analysis, when the number of instrumental variable SNPs was greater than or equal to 2, we opted for the Inverse Variance Weighted (IVW) method to estimate the potential causal effects of gut microbiota or inflammatory cytokines on ALD. In cases where the number of instrumental variable SNPs was less than 2, we employed the Wald ratio method for sensitivity analysis, a common and precise approach in MR (22). The MR results were presented as Odds Ratios (OR) and their corresponding 95% Confidence Intervals (CI). Statistical significance was considered when the P-value of IVW was less than 0.05 and the direction of the IVW estimate was consistent with the MR-Egger direction.

#### 2.4.2 Mediation analysis

In the mediation analysis, gut microbiota and inflammatory cytokines with significant causal effects on ALD from the two-sample MR analysis were included. We explored whether there was a causal relationship between gut microbiota and inflammatory cytokines (step 3, path C in **Figure 1**). If a causal effect was found between the two, multiple MR analyses would be conducted to investigate whether inflammatory cytokines act as a mediator in the pathway from gut microbiota to ALD.

#### 2.4.3 Bidirectional causal analysis

To evaluate the bidirectional causal effects among gut microbiota, inflammatory cytokines, and ALD, we used ALD as the “exposure” and gut microbiota or inflammatory cytokines associated with ALD as the “outcome” (paths a and b in **Figure 1**). SNPs significantly associated with ALD (P < 1×10^-5^) were selected as instrumental variables (IVs).

#### 2.4.4 Sensitivity analysis

To assess the robustness of the IVs, we conducted heterogeneity assessment for each SNP using Cochran’s Q test. Subsequently, we performed leave-one-out analysis by sequentially excluding each SNP and applying the IVW method to the remaining SNPs to evaluate the potential impact of specific variants on the estimates. Scatter plots of SNP-exposure associations and SNP-outcome associations, along with leave-one-out plots, were generated to visualize the MR results (23). Additionally, we employed MR-Egger intercept and MR-PRESSO methods to detect horizontal pleiotropy (24). In MR-Egger analysis, a p-value < 0.05 indicates the presence of pleiotropy (25). The MR-PRESSO method can identify potential outliers and correct for pleiotropic effects by removing these outliers.

In summary, our study utilized a two-step MR approach to assess the potential mediating role of inflammatory cytokines on the relationship between gut microbiota and ALD. We aimed to determine the microbial taxa showing a causal relationship with ALD through their impact on inflammatory cytokines. Univariable MR analyses were conducted to assess the causal effects of gut microbiota on inflammatory cytokines (Beta1), inflammatory cytokines on ALD (Beta2), and gut microbiota on ALD (Beta3). The proportion of the total effect mediated by inflammatory cytokines was estimated by dividing the indirect effect by the total effect (Beta1×Beta2/Beta3).

## 3 Results

### 3.1 Results of the weak instrumental variable test

To mitigate the bias risks stemming from weak instrumental variables, we computed the general F-statistics for each exposure factor. The F-statistics for all SNPs used as instruments for the gut microbiome exposure ranged from 19.51 to 60.95 (**Supplementary Table S1**), while the F-statistics for the SNPs used as instruments for the inflammatory cytokines exposure ranged between 19.51 and 2058.59 (**Supplementary Table S2**). Both sets of F-statistics were greater than 10, indicating a high correlation between the instrumental variables and the exposure factors (26).

### 3.2 Causal relationships between the gut microbiome, inflammatory cytokines and ALD

#### 3.2.1 Impact of the gut microbiome on ALD

The IVW MR analysis revealed significant causal relationships between ALD and three specific gut microbial taxa: *Escherichia coli* (OR= 0.69, 95% CI: 0.54-0.88, *P*= 0.003), *Roseburia hominis* (OR=1.50, 95% CI: 1.06-2.14, *P*=0.023), and the bacterial *Family Porphyromonadaceae* (OR=1.30, 95% CI: 1.01-1.66, *P*=0.038). The results showed that *Escherichia coli* had a negative causal relationship with ALD (OR < 1), suggesting a protective effect of this gut microbiome component against the development of ALD (**Table 1**). On the other hand, *Roseburia hominis* and *the bacterial Family Porphyromonadaceae* showed positive causal relationships with ALD (OR > 1), indicating they may act as risk factors for ALD development (**Table 1**).

**Table 1:** Mendelian randomization analysis on the causal effect between gut microbiota and ALD.

Gut microbiota	Methods	IVs	P value	OR	95% CI	Egger intercept, p value	Heterogeneity (Q, p value)
Escherichia_coli	Inverse variance weighted	12	0.0028	0.689	0.69 (0.54-0.88)	0.037, 0.460	15.688, 0.403
	Weighted median	12	0.0879	0.760	0.76 (0.55-1.04)		
	Simple mode	12	0.5698	0.847	0.85 (0.49-1.47)		
	MR Egger	12	0.7180	1.204	1.20 (0.45-3.21)		15.066, 0.374
	Weighted mode	12	0.7238	0.907	0.91 (0.53-1.54)		
Roseburia_hominis	Inverse variance weighted	7	0.0233	1.503	1.50 (1.06-2.14)	-0.073, 0.277	15.728, 0.152
	Weighted median	7	0.1026	1.441	1.44 (0.93-2.24)		
	Simple mode	7	0.1250	1.816	1.82 (0.94-3.50)		
	Weighted mode	7	0.5718	1.227	1.23 (0.63-2.40)		
	MR Egger	7	0.7171	1.938	1.94 (0.07- 57.01)		13.890, 0.178
Porphyromonadaceae	Inverse variance weighted	16	0.0376	1.297	1.30 (1.01-1.66)	-0.023, 0.888	1.876, 0.931
	Weighted median	16	0.0499	1.399	1.40 (1.00-1.96)		
	Weighted mode	16	0.2180	1.430	1.43 (0.83-2.47)		
	Simple mode	16	0.2550	1.422	1.42 (0.79-2.55)		
	MR Egger	16	0.8264	0.893	0.89 (0.33-2.41)		1.854, 0.869

IV, instrumental variables; OR, Odd Ratio; 95%CI, 95% confidence intervals.

**Figure 2:**
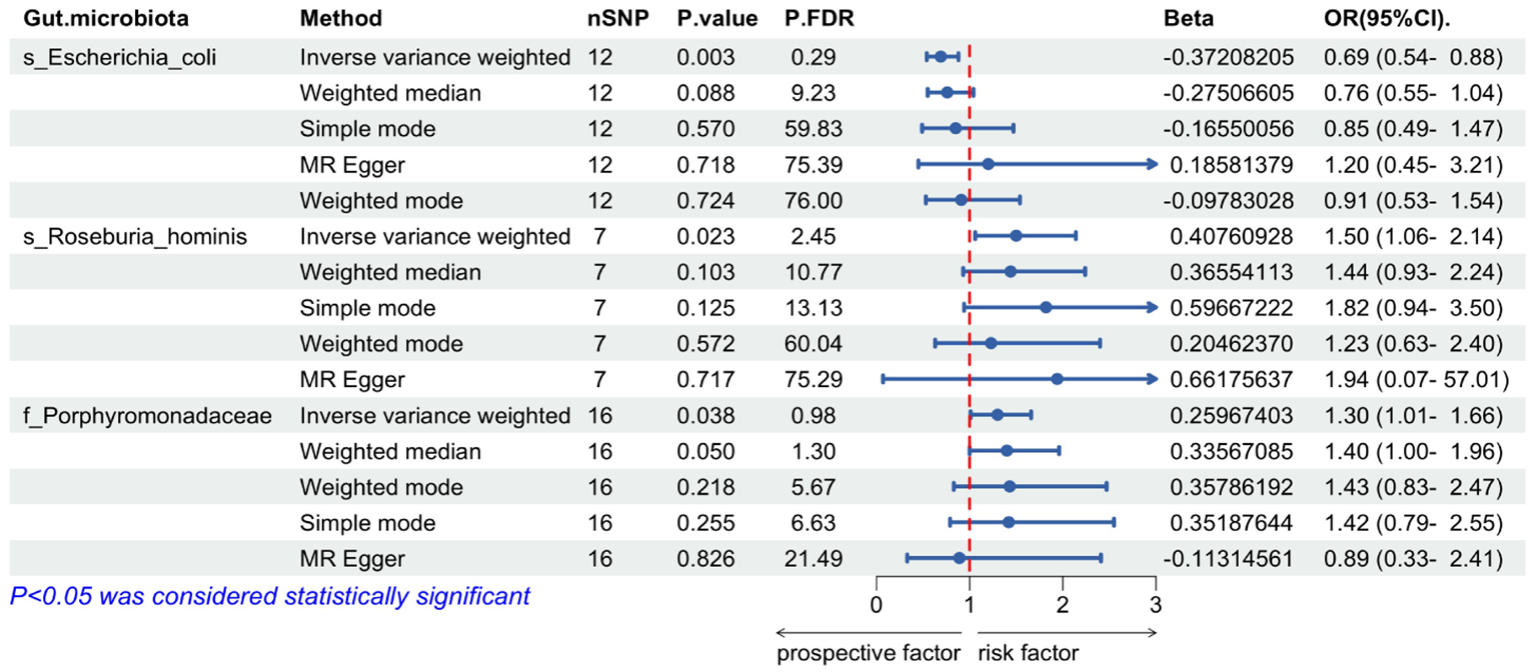
Mendelian randomization of causal effects between gut microbiota and ALD. P.FDR is the p-value adjusted for multiple comparisons using the False Discovery Rate (FDR) method; nSNP refers to the number of genetic variants used as instrumental variables in the MR analysis; Beta refers to the estimated causal effect of the exposure on the outcome.

Through the MR-Egger regression intercept method, the analysis found no evidence of bias due to genetic pleiotropy in the results. Cochran’s Q test indicated there was no statistically significant heterogeneity (P > 0.05), and the MR-PRESSO analysis revealed no horizontal pleiotropy in this MR study (**Figure 2**).

#### 3.2.2 Influence of inflammatory cytokines on ALD

In our IVW MR analysis of the impact of inflammatory cytokine levels on ALD, we identified seven inflammatory cytokines whose genetically-predicted expressions were significantly causally related to ALD. Among them, five cytokines showed positive causal relationships with ALD——CUB domain-containing protein 1(OR = 1.16, 95% CI: 1.01–1.32, P = 0.035), Macrophage colony-stimulating factor 1(OR = 1.23, 95% CI: 1.00–1.51, P = 0.047), Cystatin D (OR= 1.09, 95%CI: 1.01-1.18, P= 0.035), Fractalkine (OR=1.53, 95%CI: 1.32-1.78, P=0.000000038), Monocyte chemoattractant protein-1 (OR=1.24, 95%, CI: 1.07-1.44, P=0.004),suggesting their potential to increase the risk of ALD (**Table 2**). Conversely, the genetically-predicted expressions of two inflammatory cytokines, CD40L receptor(OR = 0.89, 95%CI: 0.794–0.997, P = 0.044), Leukemia inhibitory factor(OR = 0.73, 95%CI: 0.55–0.96, P = 0.024)exhibited negative causal relationships with ALD, indicating their potential to reduce the risk of ALD (**Table 2**).

**Table 2:** Mendelian randomization analysis on the causal effect between inflammatory cytokines and ALD.

inflammatory cytokines	Methods	IVs	P value	OR	95% CI	Egger intercept, p value	Heterogeneity (Q, p value)
CD40 measurement	MR Egger	49	0.4045	0.921	0.92 (0.76-1.11)	0.037, 0.460	58.016, 0.296
	Weighted median	49	0.4655	0.940	0.94 (0.80-1.11)		
	Inverse variance weighted	49	0.0442	0.890	0.89 (0.79-1.00)		58.134, 0.326
	Simple mode	49	0.2173	0.834	0.83 (0.63-1.11)		
	Weighted mode	49	0.2666	0.914	0.91 (0.78-1.07)		
CUB domain-containing protein 1 measurement	MR Egger	64	0.1272	1.261	1.26 (0.94-1.69)	-0.006, 0.645	63.370, 0.806
	Weighted median	64	0.1352	1.157	1.16 (0.96-1.40)		
	Inverse variance weighted	64	0.0346	1.157	1.16 (1.01-1.32)		63.584, 0.824
	Simple mode	64	0.4595	1.162	1.16 (0.78-1.73)		
	Weighted mode	64	0.5469	1.099	1.10 (0.81-1.49)		
macrophage colony-stimulating factor 1 measurement	MR Egger	29	0.6263	1.127	1.13 (0.70-1.81)	-0.012, 0.608	29.436, 0.597
	Weighted median	29	0.4280	1.133	1.13 (0.83-1.54)		
	Inverse variance weighted	29	0.0466	1.230	1.23 (1.00-1.51)		29.704, 0.632
	Simple mode	29	0.1003	1.730	1.73 (0.92-3.26)		
	Weighted mode	29	0.7690	1.055	1.05 (0.74-1.50)		
cystatin-D measurement	MR Egger	97	0.7243	1.023	1.02 (0.90-1.16)	0.007, 0.402	82.027, 0.953
	Weighted median	97	0.2050	1.081	1.08 (0.96-1.22)		
	Inverse variance weighted	97	0.0350	1.090	1.09 (1.01-1.18)		82.736, 0.954
	Simple mode	97	0.4454	1.092	1.09 (0.87-1.37)		
	Weighted mode	97	0.3068	1.067	1.07 (0.94-1.21)		
fractalkine measurement	MR Egger	57	0.5402	1.102	1.10 (0.81-1.50)	0.036, 0.019	64.199, 0.434
	Weighted median	57	0.0003	1.536	1.54 (1.22-1.94)		
	Inverse variance weighted	57	0.0000	1.530	1.53 (1.31-1.78)		70.112, 0.280
	Simple mode	57	0.0357	1.764	1.76 (1.05-2.96)		
	Weighted mode	57	0.0105	1.552	1.55 (1.12-2.15)		
leukemia inhibitory factor measurement	MR Egger	21	0.7052	1.136	1.14 (0.59-2.17)	-0.024, 0.337	24.274, 0.560
	Weighted median	21	0.1032	0.724	0.72 (0.49-1.07)		
	Inverse variance weighted	21	0.0242	0.728	0.73 (0.55-0.96)		25.231, 0.562
	Simple mode	21	0.1436	0.623	0.62 (0.34-1.15)		
	Weighted mode	21	0.1882	0.672	0.67 (0.38-1.19)		
CCL2 measurement	MR Egger	39	0.7710	1.044	1.04 (0.78-1.39)	0.000, 0.999	52.450, 0.271
	Weighted median	39	0.0039	1.352	1.35 (1.10-1.66)		
	Inverse variance weighted	39	0.0043	1.242	1.24 (1.07-1.44)		52.450, 0.306
	Simple mode	39	0.0442	1.438	1.44 (1.02-2.03)		
	Weighted mode	39	0.0463	1.320	1.32 (1.01-1.72)		

IV, instrumental variables; OR, Odd Ratio; 95%CI, 95% confidence intervals.

**Figure 3:**
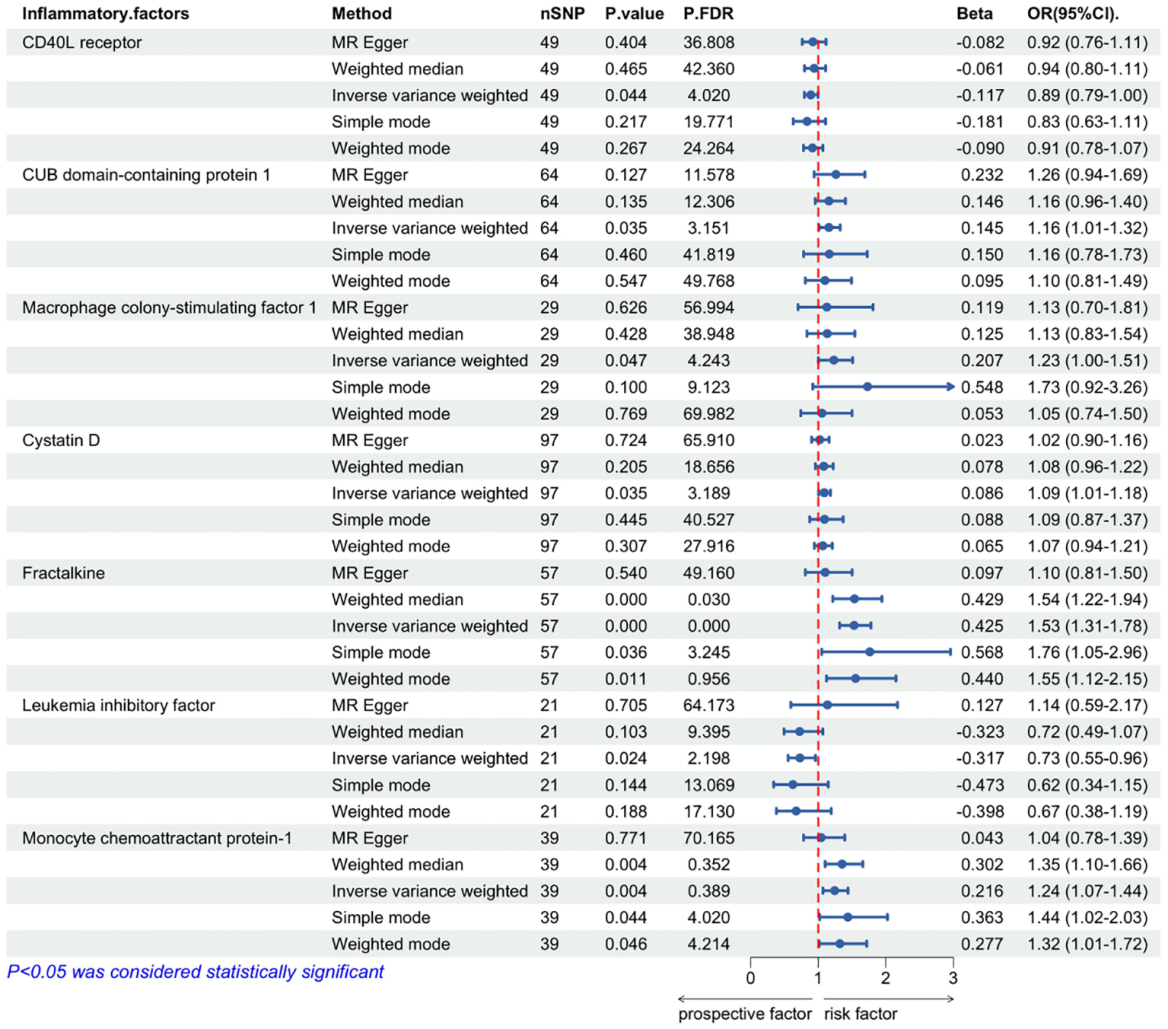
Mendelian randomization of causal effects between inflammatory cytokines and ALD. P.FDR is the p-value adjusted for multiple comparisons using the False Discovery Rate (FDR) method; nSNP refers to the number of genetic variants used as instrumental variables in the MR analysis; Beta refers to the estimated causal effect of the exposure on the outcome.

To validate these results, sensitivity and pleiotropy analyses were conducted using MR-Egger and Cochran’s Q test methods, which confirmed the absence of significant heterogeneity in the results. Additionally, MR-PRESSO analysis showed P>0.05, indicating no evidence of horizontal pleiotropy in the MR analysis (**Figure 3**).

#### 3.2.3 Influence of gut microbiota on inflammatory cytokines

In the preceding univariate MR analyses, a total of three gut microbiota species and seven inflammatory cytokines were found to have significant causal relationships with ALD, without evidence of heterogeneity or horizontal pleiotropy. Subsequently, we conducted two-sample MR analyses between these three gut microbiota species and seven inflammatory cytokines. The results revealed that two gut microbial species were causally related to four inflammatory cytokines. Specifically, only the genetically-predicted expression of CUB domain-containing protein 1 (OR = 1.07, 95% CI: 1.01–1.13, *P* = 0.032) showed a positive causal relationship with *Escherichia coli*, indicating that an increase in *Escherichia coli* may enhance the expression levels of CUB domain-containing protein 1. Additionally, four inflammatory cytokines were found to have causal relationships with the gut microbiome component *Roseburia hominis*: CD40L receptor (OR = 1.21, 95% CI: 1.07–1.36, P = 0.002), CUB domain-containing protein 1 (OR = 1.12, 95% CI: 1.01–1.24, P = 0.035), Cystatin D (OR = 1.19, 95% CI: 1.04–1.37, P = 0.012), and Monocyte chemoattractant protein-1 (OR = 1.17, 95% CI: 1.05–1.30, *P*= 0.005) (**Table 3**).

**Table 3:** Mendelian randomization analysis on the causal effect between gut microbiota and inflammatory cytokines.

Gut microbiota - inflammatory cytokines	Methods	IVs	P value	OR	95% CI	Egger intercept, p value	Heterogeneity (Q, p value)
Escherichia_coli-CUB domain-containing protein 1	MR Egger	12	0.4260	0.897	0.90 (0.70-1.16)	0.023, 0.199	4.303, 0.932
	Weighted median	12	0.1221	1.065	1.06 (0.98- 1.15)		
	Inverse variance weighted	12	0.0316	1.068	1.07 (1.01-1.13)		6.190, 0.860
	Simple mode	12	0.3321	1.066	1.07 (0.94-1.20)		
	Weighted mode	12	0.3828	1.063	1.06 (0.93-1.21)		
Roseburia_hominis-CD40L receptor	MR Egger	7	0.0416	3.904	3.90 (1.47-10.40)	-0.108, 0.065	2.369, 0.796
	Weighted median	7	0.0151	1.206	1.21 (1.04-1.40)		
	Inverse variance weighted	7	0.0023	1.207	1.21 (1.07-1.36)		7.948, 0.242
	Simple mode	7	0.1641	1.228	1.23 (0.97-1.58)		
	Weighted mode	7	0.1384	1.217	1.22 (0.97-1.53)		
Roseburia_hominis-CUB domain-containing protein 1	MR Egger	7	0.9435	0.964	0.96 (0.36-2.55)	0.014, 0.777	0.884, 0.971
	Weighted median	7	0.1084	1.113	1.11 (0.97-1.27)		
	Inverse variance weighted	7	0.0353	1.117	1.12 (1.01-1.24)		0.974, 0.987
	Simple mode	7	0.1426	1.154	1.15 (0.98-1.36)		
	Weighted mode	7	0.3171	1.102	1.10 (0.93-1.31)		
Roseburia_hominis-Cystatin D	MR Egger	7	0.9572	1.041	1.04 (0.26-4.24)	0.012, 0.858	10.112, 0.072
	Weighted median	7	0.0404	1.167	1.17 (1.01-1.35)		
	Inverse variance weighted	7	0.0123	1.191	1.19 (1.04-1.37)		10.184, 0.117
	Simple mode	7	0.2119	1.177	1.18 (0.94-1.48)		
	Weighted mode	7	0.1932	1.169	1.17 (0.95-1.44)		
Roseburia_hominis-Monocyte chemoattractant protein-1	MR Egger	7	0.6671	1.262	1.26 (0.46-3.43)	-0.007, 0.882	0.848, 0.974
	Weighted median	7	0.0149	1.176	1.18 (1.03-1.34)		
	Inverse variance weighted	7	0.0046	1.166	1.17 (1.05-1.30)		0.873, 0.990
	Simple mode	7	0.0957	1.205	1.21 (1.00-1.45)		
	Weighted mode	7	0.1107	1.201	1.20 (0.99-1.46)		

IV, instrumental variables; OR, Odd Ratio; 95%CI, 95% confidence intervals.

#### 3.2.4 Mediating role of inflammatory cytokines in the gut microbiota-ALD causal pathway

Within the aforementioned five pathways involving gut microbiota, inflammatory cytokines, and ALD causality, we also calculated the Mediated Proportion of these inflammatory cytokines. Through further analysis, we found that there were three mediatory pathways (Beta1×Beta2) that were consistent with the direction of the gut microbiota-ALD causal relationship (Beta3), and all of these mediatory pathways were related to the gut microbiome component *Roseburia hominis*. *Roseburia hominis* was found to increase the risk of ALD by mediating the levels of the risk factors CUB domain-containing protein 1, Cystatin D, or Monocyte chemoattractant protein-1. This suggests that in MR analysis, *Roseburia hominis* may promote the onset of ALD through the mediation of multiple inflammatory cytokines, indicating its potential key role in the development of ALD.

### 3.3 Bidirectional causal effects between ALD, gut microbiota, and inflammatory cytokines

There were no opposing causal effects detected between gut microbiota, inflammatory cytokines, and ALD. After matching the Functional Training Dataset (FTD) with gut microbiota or cytokines, no single nucleotide polymorphism (SNP) could be used as a valid instrumental variable.

## 4 Discussion

In the discussion, as far as we know, this study is the first to employ MR analysis to estimate the causal relationship between gut microbiota and ALD, as well as the mediating role of inflammatory cytokines. In this comprehensive mediating MR analysis, we identified multiple gut microbiota taxa that play crucial roles in the development of ALD, with three gut microbiota taxa (*Escherichia coli*, *Roseburia hominis*, *Family Porphyromonadaceae*) showing a causal relationship with ALD. The mediating MR results indicate that among these three microbiota taxa, only *Roseburia hominis* is involved in mediating causal pathways with inflammatory cytokines. Specifically, three inflammatory cytokines, including CUB domain-containing protein 1, Cystatin D, or Monocyte chemoattractant protein-1, may respectively account for 1.6%, 1.5%, and 3.3% of the impact of *Roseburia hominis* on ALD. Furthermore, all three types of inflammatory cytokines are risk factors for the occurrence and progression of ALD. This analysis highlights the connection between gut microbiota and ALD, emphasizing the mediating role of inflammatory cytokines. In addition, ALD itself may not affect changes in gut microbiota and inflammatory cytokines that we have described. This suggests that changes in the gut microbiota and inflammatory cytokines occur prior to the onset of ALD, rather than being a consequence of the disease process.

In most cases of chronic liver diseases, dysbiosis of gut microbiota serves as the cornerstone of gut-liver axis impairment. Changes in gut-liver axis characteristics in ALD patients can lead to compromised intestinal integrity, disrupted bile acid metabolism, translocation of pathogen-associated molecular patterns (PAMPs), live microbes, and microbial metabolites, further exacerbating liver inflammation (4, 10). Dysbiosis of gut microbiota results in intestinal inflammation, ethanol metabolites, intestinal bile acids, and potentially other metabolites, all contributing to the breakdown of the intestinal barrier, increasing susceptibility to ALD, and compromising the patient’s health (27). *Roseburia hominis* (*R. hominis*) is a representative species within the Roseburia genus, belonging to the Lachnospiraceae family within the Firmicutes phylum. Recent studies have shown that *R. hominis* can metabolize various short-chain fatty acids (SCFAs) including acetate, propionate, and butyrate, which are considered potential therapeutic factors in the treatment of neuroinflammation and colitis (28, 29).

Experiments by Patterson AM et al. suggested that the metabolites of *R. hominis* in the gut, particularly SCFAs, have multifaceted regulatory effects on gastrointestinal diseases (30). Through oral administration to colitis mice for 14 days, Patterson AM found that the group receiving *R. hominis* had lower levels of colonic mucosal inflammation compared to the untreated colitis mice, indicating a positive correlation between increased levels of *R. hominis* and reduced colonic mucosal inflammation. In a study by Song L et al. involving male Sprague-Dawley germ-free rats, oral administration of *R. hominis* resulted in a significant increase in melatonin levels in the gut. Interestingly, there was a significant increase in the concentrations of propionate and butyrate in the gut contents after gavage (31). In another study by the same team on the inhibitory effects of *R. hominis* on neuroinflammation, they also identified the presence of propionate and butyrate, derivatives of *R. hominis* (28). As *R. hominis* entered the bodies of germ-free rats through oral administration, the abundance of propionate and butyrate increased, leading to reduced activation of microglial cells in the hippocampus and decreased release of pro-inflammatory cytokines and chemokines. This suggests that *R. hominis* may improve gut inflammation and neuroinflammation through its derivatives, positioning *R. hominis* as a “multifunctional probiotic” in human life. This seems to contradict our analysis results that “*R. hominis* may exacerbate ALD through inflammatory factors”.

However, in a study on the correlation between gut microbiota composition at disease onset and major adverse cardiovascular events within 3.2 years, the molar ratio of acetate and butyrate in the feces of acute myocardial infarction (AMI) patients was higher, contradicting the role of SCFAs in intestinal inflammation (32). Concurrently, research by Ji et al. found that a high abundance of Romboutsia and Roseburia not only failed to improve symptoms in Parkinson’s disease patients but was also associated with an increased risk of Parkinson’s disease (33). These findings challenge the “probiotic” identity of *R. hominis*, as it and its derivatives appear to have varying effects on the human body. To our knowledge, no team has investigated the relationship between *R. hominis* and ALD or even liver diseases beyond its derivatives. Our research results suggest a causal relationship between the gut microbiome composition, particularly *R. hominis*, and the development of ALD. To explore potential mediating pathways, we further analyzed the mediating effects of inflammatory cytokines.

The CUB domain-containing protein 1 (CDCP1) is a type I transmembrane glycoprotein that is widely upregulated in the pathogenesis of various malignant tumors, including those of the liver and pancreas (34). CDCP1 serves as a marker for tumor progression and also accelerates tumor metastasis. Stimulation of CDCP1 expression promotes CDCP1-mediated cancer cell migration *in vitro* and exacerbates the pro-carcinogenic effects, possibly by promoting the formation of a tumor inflammatory microenvironment (35, 36). In studies related to non-alcoholic steatohepatitis (NASH), a significant decrease in circulating CDCP1 was observed after weight loss surgery, and CDCP1 levels showed a close correlation with liver injury markers ALT and AST, suggesting CDCP1 may be a risk factor for NASH (37). Our research indicates a positive correlation between CDCP1 and ALD, with CDCP1 being a risk factor for the onset of ALD. Combining with previous studies, we infer that CDCP1 may promote the transformation of ALD into alcohol-related liver cancer.

Cystatin D (CST5) belongs to cystatin family II, which is from the cystatin superfamily. Studies have shown that CST5 profoundly affects cell phenotypes, increasing cell adhesion and inhibiting cell proliferation and migration. Researchers have observed that the expression of CST5 in human colon cancer cells is the opposite of CDCP1, significantly reducing the tumorigenic potential in immunodeficient mice (38). However, in hepatitis B virus-related liver cancer, the levels of CST5 in tumors are lower than in normal tissues (39). Our analysis results show that CST5 is also a risk factor for disease progression in ALD, suggesting that the role and mechanisms of CST5 may vary in different organs, and it may play a detrimental role in liver diseases.

Monocyte chemoattractant protein-1(MCP-1) is a member of the CC chemotaxis family, also known as Chemokine (CC-motif) ligand 2 (CCL2). MCP-1 is widely implicated in various diseases, including NASH, neuroinflammatory disorders, rheumatoid arthritis, cardiovascular diseases, and cancer, playing a role in attracting or enhancing the expression of other inflammatory factors and cells during these processes (40, 41). MCP-1 can recruit additional liver cells by regulating inflammatory cells, leading to liver fibrosis. Moreover, MCP-1 can also induce liver cell necrosis, fibronectin deposition, and DNA changes by modulating the expression of immune cells, contributing to the occurrence and development of liver tumors and liver cancer (42). In studies related to ALD, Umhau et al. conducted a multiple regression analysis of MCP-1 levels in the cerebrospinal fluid of alcoholics and the liver enzymes gamma-glutamyl transferase (GGT) and aspartate aminotransferase/glutamic-oxaloacetic transaminase (AST/GOT) in the blood, finding a positive correlation between MCP-1 and GGT as well as AST/GOT (43). This suggests a close association between MCP-1 and ALD, with MCP-1 potentially being a risk factor for the development of ALD. The researchers’ genetic validation confirmed a causal relationship between the two. However, further research is needed to elucidate the mechanisms by which MCP-1 contributes to ALD.

In the analysis process, we utilized a variety of common sensitivity analyses and excluded the influence of confounding factors and reverse causation. The preliminary research findings suggest a causal relationship between gut microbiota and ALD, as well as the involvement of inflammatory cytokines, providing further theoretical support and new directions for the treatment and prevention of ALD. Interestingly, the data analysis revealed that one gut bacterium (*R. hominis*) can regulate ALD by modulating multiple inflammatory cytokines (CDCP1, CST5, MCP-1). Although the individual effects of these inflammatory cytokines are relatively low, they offer a research path for the multi-target regulation of ALD by a single gut bacterium.

While the study has many strengths, there are some limitations. Firstly, the participants were exclusively of European descent, which limit the generalizability of the findings to other regions or ethnicities. Secondly, the results are based on theoretical data analysis and have not been validated through clinical or animal experiments, necessitating further elucidation through additional cellular, animal, and clinical studies. Nonetheless, the present study leveraged the largest available data collection, utilizing the most recently curated data source, and involving a substantial number of participants. Furthermore, rigorous sensitivity analyses have demonstrated the stability of our results, lending a high degree of reliability to the findings. Thirdly, apart from the 91 inflammatory cytokines studied, there may be other cytokines that were not included in this research, indicating the possibility of additional cytokines participating in the causal effects of gut microbiota on ALD (44). Lastly, although three inflammatory cytokines that mediate the causal relationship between gut microbiota and ALD have been identified and analyzed, their specific mechanisms in influencing the progression of ALD still warrant further investigation.

## 5 Conclusion

In this study, we comprehensively explored the causal effects between gut microbiota, inflammatory cytokines, and ALD. There were two positive and one negative causal effect between genetic liability in the gut microbiota and ALD. There were five positive correlations and two negative causal effects between inflammatory cytokines and ALD. In addition, we found one type of gut microbiota can separately regulate ALD through three different inflammatory cytokines.

## Data Availability

The original contributions presented in the study are included in the article/**Supplementary Material**. Further inquiries can be directed to the corresponding author.
